# Quantification of HEV RNA by Droplet Digital PCR

**DOI:** 10.3390/v8080233

**Published:** 2016-08-19

**Authors:** Florence Nicot, Michelle Cazabat, Sébastien Lhomme, Olivier Marion, Karine Sauné, Julie Chiabrando, Martine Dubois, Nassim Kamar, Florence Abravanel, Jacques Izopet

**Affiliations:** 1 CHU Toulouse, Hôpital Purpan, Laboratoire de virologie, Institut fédératif de biologie, Toulouse F-31300, France; cazabat.m@chu-toulouse.fr (M.C.); lhomme.s@chu-toulouse.fr (S.L.); saune.k@chu-toulouse.fr (K.S.); chiabrando.j@chu-toulouse.fr (J.C.); dubois.m@chu-toulouse.fr (M.D.); abravanel.f@chu-toulouse.fr (F.A.); izopet.j@chu-toulouse.fr (J.I.); 2 INSERM, U1043, Centre de Physiopathologie de Toulouse Purpan, Toulouse F-31300, France; marion-olivier@hotmail.fr (O.M.); kamar.n@chu-toulouse.fr (N.K.); 3 Université Toulouse III Paul-Sabatier, Faculté de Médecine Toulouse, Toulouse F-31300, France; 4 CHU Toulouse, Hôpital Rangueil, Département de Néphrologie, Dialyse et Transplantation multi-organe, Toulouse F-31300, France

**Keywords:** ddPCR, absolute quantification, HEV quantification

## Abstract

The sensitivity of real-time PCR for hepatitis E virus (HEV) RNA quantification differs greatly among techniques. Standardized tools that measure the real quantity of virus are needed. We assessed the performance of a reverse transcription droplet digital PCR (RT-ddPCR) assay that gives absolute quantities of HEV RNA. Analytical and clinical validation was done on HEV genotypes 1, 3 and 4, and was based on open reading frame (ORF)3 amplification. The within-run and between-run reproducibilities were very good, the analytical sensitivity was 80 HEV RNA international units (IU)/mL and linearities of HEV genotype 1, 3 and 4 were very similar. Clinical validation based on 45 samples of genotype 1, 3 or 4 gave results that correlated well with a validated reverse transcription quantitative PCR (RT-qPCR) assay (Spearman *r_s_* = 0.89, *p* < 0.0001). The RT-ddPCR assay is a sensitive method and could be a promising tool for standardizing HEV RNA quantification in various sample types.

## 1. Introduction

Hepatitis E virus (HEV) is a non-enveloped single-stranded, positive sense RNA virus [1]. Four main genotypes have been identified, of which genotype 3 predominates in industrialized countries [2,3,4,5]. HEV genotype 3 is of increasing concern for immunocompromised patients as it can lead to chronic infection and cirrhosis [6,7]. An HEV infection is best diagnosed by a combination of serological tests and nucleic acid amplification techniques [1]. An accurate quantitative assay for HEV RNA is needed for pathophysiological studies and for monitoring the HEV viral loads of chronically infected patients on antiviral therapy [8,9]. An evaluation of several reverse transcriptase quantitative PCR (RT-qPCR) methods showed that their sensitivities differed greatly [10].

We have evaluated a new method for quantification of HEV RNA, reverse transcriptase droplet digital PCR (RT-ddPCR), that gives absolute quantification of the target gene [11,12].

## 2. Materials and Methods

HEV-positive plasma samples whose virus content had been quantified by a validated RT-qPCR method (accreditation ISO 15189) was used to assess the analytical performance of the reverse transcription droplet digital PCR (RT-ddPCR) [13]. Both methods target the same region (a 70 bp sequence in the open reading frame (ORF3) region) using a forward primer HEVORF3-S (5′-GGTGGTTTCTGGGGTGAC-3′), a reverse primer HEVORF3-AS (5′-AGGGGTTGGTTGGATGAA-3′), and the probe 5′–6-carboxyfluorescein (FAM)–TGATTCTCAGCCCTTCGC–6-carboxytetramethylrhodamine (TAMRA)–3′. HEV RNA was extracted from plasma samples (140 µL) with the RNeasy minikit (Qiagen, Courtaboeuf, France) according to the manufacturer’s instruction. The 50 µL reaction mix of RT-qPCR contained 1 µL of SuperScript III Platinum One-Step quantitative RT-PCR system medium (Invitrogen by Life Technologies Corporation, Carlsbad, CA, USA), 20 µL of RNA, primers (200 nM), probes (150 nM), and 40 U/reaction RNase Out (Invitrogen). The 20 µL RT-ddPCR reaction mixture contained 10 µL of One-step RT-ddPCR mix for Probes (BioRad, Pleasanton, CA, USA), HEV primers (500 nM)/probes (250 nM) and 7 µL nucleic acid. Reverse transcription was carried out at 61 °C for 30 min followed by denaturation at 95 °C for 5 min, and DNA was amplified with 40 PCR cycles at 95 °C (30 s) and 56 °C (1 min) on a QX200 droplet digital PCR system (BioRad). After PCR, read-out of positive versus negative droplets was performed with the droplet reader and the absolute quantification of PCR target was analysed using QuantaSoft software (BioRad). The RT-ddPCR assay was compared to a validated RT-qPCR method [13]. Clinical specimens from 45 HEV individuals infected with genotype 1 (*n* = 4), genotype 3 (15 subtypes 3c/3i and 16 subtypes 3e/3f) and genotype 4 (*n* = 10) were tested by both methods. Statistical analyses were performed with XLSTAT software (version 14, Addinsoft, Paris, France).

## 3. Results

### 3.1. Analytical Performance of the RT-ddPCR Assay

The limit of detection was determined using serial dilutions of the international standard (Paul Ehrlich Institute, Langen, Hessen, Germany; genotype 3a) [14] in HEV-negative plasma to give 1000 (5 replicates tested), 200, 100, 50 and 20 HEV RNA international unit (IU)/mL (15 replicates of each concentration tested). Probit analysis predicted a limit of detection (LOD) of 80 (95% confidence interval: 61–156) HEV RNA IU/mL. Negative plasma samples were tested 26 times in 15 independent series. All RT-ddPCR wells were negative. The risk of cross-contamination was assessed by testing 12 negative samples alternating with positive samples (6 log copies/mL) in the same assay. None of the negative samples gave a positive signal. The within-run reproducibility was evaluated with eight replicates of HEV RNA extracted from a plasma sample that contained 4.7 log copies/mL. The within-run standard deviation (SD) was 0.05 and the coefficient of variation (CV) was 0.97% for a mean value of 4.7 log copies/mL. Between-runs reproducibility was evaluated with the international standard diluted to give 3.1 log copies/mL. It was tested in six independent RT-ddPCR series. The between-runs SD was 0.1 and the CV was 4% for a mean value of 3.3 log copies/mL. We calculated the dynamic range of the method by testing triplicates of serial dilutions of HEV genotype 1, genotype 3 and genotype 4 positive plasma (10^6^ to 200 copies/mL). HEV genotype 2 was not tested due to the absence of available samples. The observed mean and SD for each concentration are listed in Table 1. Regression analysis gave *y* = 1.008*x* − 0.1002 with a *R^2^* = 0.990 for genotype 1, *y* = 1.028*x* − 0.1275 with a *R^2^* = 0.998 for genotype 3 and *y* = 0.9564*x* − 0.1495 with a *R^2^* = 0.986 for genotype 4. Concentrations above 10^6^ copies/mL were not analysable; sample dilution was mandatory.

### 3.2. Comparison of HEV RNA Quantification between RT-ddPCR and RT-qPCR

**Figure 1:**
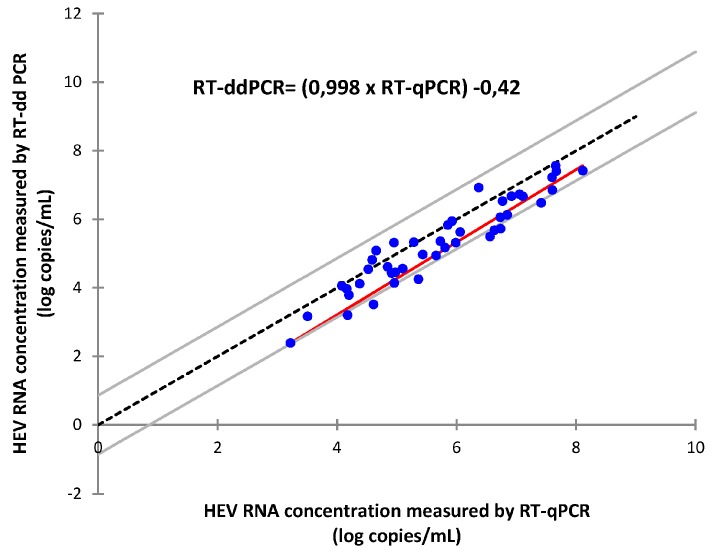
Passing-Bablock regression analysis of hepatitis E virus (HEV) RNA concentrations determined by reverse transcriptase droplet digital PCR (RT-ddPCR) and reverse transcriptase quantitative PCR (RT-qPCR) assays (*n* = 43). The red line represents the Passing-Bablock regression line, the dashed line represents the identity line and grey lines represent the confidence interval for the regression line. Dots represent HEV RNA concentrations measured by RT-qPCR and RT-ddPCR for each sample.

**Figure 2:**
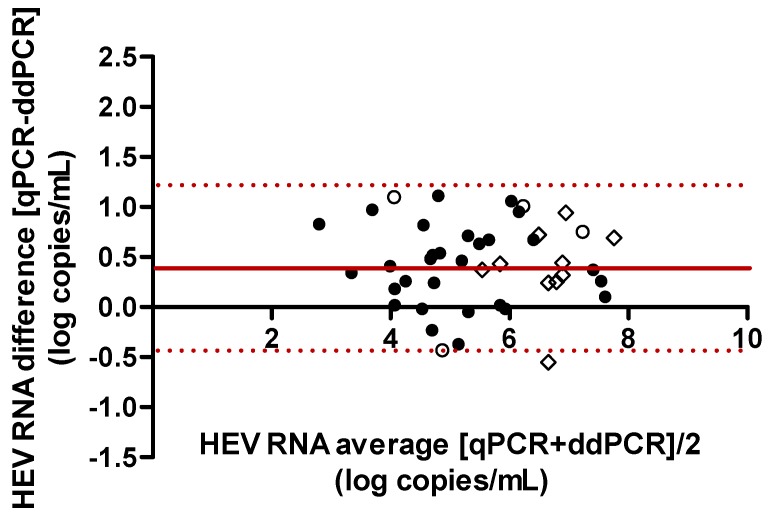
Agreement between HEV RNA concentrations measured by RT-qPCR and RT-ddPCR. Bland-Altman plots comparing HEV RNA quantified by RT-qPCR and RT-ddPCR. Solid lines show the mean of the differences (*n* = 0.42 log HEV RNA copies/mL) and broken lines show the mean ± 2 standard deviation. Empty circles designate genotype 1, full circles indicate genotype 3 and squares indicate genotype 4. The two samples not assayed by RT-ddPCR were excluded from the Bland-Altman analysis.

Plasma samples from 45 individuals infected with HEV were tested by both methods (samples with HEV RNA concentrations above 6 log copies/mL were diluted 1/100). Passing-Bablock regression analysis defined RT-ddPCR as ((0.998 × RT-qPCR) − 0.42) (Figure 1). Bland-Altman analysis indicated that RT-qPCR gave a slightly higher virus load than did RT-ddPCR, with an average difference of 0.42 log copies/mL. Thus the RT-qPCR method probably overestimates the HEV RNA concentration (Figure 2). The correlation between the two methods was good (Spearman *r_s_* = 0.89, *p* < 0.0001). Two samples (1 subtype 3e and 1 subtype 3c) were not quantifiable by RT-ddPCR.

## 4. Discussion

Our data therefore indicate that RT-ddPCR performs well when used to quantify HEV genotypes 1, 3 and 4. The between-runs reproducibility was similar to those obtained for the RT-qPCR methods based on ORF3 (SD = 0.15–0.21) and the sensitivity is comparable to that of real-time PCR methods based on ORF3 (20–100 HEV RNA IU/mL) [13,15]. Different genotypes of HEV RNA can be quantified by the RT-ddPCR with similar dynamic range. Sequence alignment of primer/probes with HEV reference sequences show none or only one mismatch in a non-critical position among genotypes 1–7 (data not shown). This deals with their capacity to detect HEV strains representing genotypes 1 to 4 by real-time PCR assays [16].

One limitation of the ddPCR is that samples containing a high concentration of target molecules must be diluted [12,17,18]. The response is not linear at high concentrations because the system can no longer discriminate between positive and negative droplets. The samples assayed in the present study that had HEV RNA concentrations above 6 log copies/mL could be accurately quantified only after dilution. Our RT-qPCR assays showed that the two samples not quantifiable by RT-ddPCR had high HEV RNA concentrations: 6.73 and 7.66 log copies/mL. Positive and negative droplets were not distinguishable even after 100-fold dilution. This problem was probably artefactual as it was possible to quantify all other samples with high concentrations after dilution. A problem with primers or probe was discarded because the RT-qPCR method used the same primers/probe. We could not exclude a problem of sample matrix with these two samples, hampering a correct analysis.

The ddPCR method gives absolute quantities of HEV RNA; it does not require a standard curve. Standard curves are often generated with plasmids or transcripts, which can lead to differences in quantification obtained with various RT-qPCR assays. This could well explain the overestimation observed with RT-qPCR compared to RT-ddPCR. The RT-ddPCR could be used to generate a calibrated panel of HEV strains and to evaluate more precisely the sensitivities of nucleic acid test assays for HEV RNA, which greatly differ among techniques [10,19]. The ddPCR assay is therefore a promising tool for standardizing HEV RNA quantification not only in blood but also in food products and water samples.

## Figures and Tables

**Table 1:** Linearity of hepatitis E virus (HEV) RNA quantification by reverse transcriptase droplet digital PCR (RT-ddPCR). Each concentration was tested in triplicate.

Plasma Dilution (HEV RNA Copies/mL)	Replicate 1 Replicate 2 Replicate 3 (HEV RNA Log Copies/mL)			Mean (HEV RNA Log Copies/mL)	SD
**Genotype 1**					
**1,000,000**	5.90	5.90	5.91	5.90	0
**100,000**	5	5	4.99	5	0.01
**10,000**	3.99	4.12	4.05	4.05	0.06
**1000**	2.01	3.05	3.01	2.69	0.59
**500**	2.44	2.61	2.61	2.55	0.10
**200**	2.68	2.47	2.01	2.39	0.34
**Genotype 3**					
**1,000,000**	6.05	6	5.99	6.01	0.03
**100,000**	5	5.01	5.02	5.01	0.01
**10,000**	4.02	4.04	4.03	4.03	0.01
**1000**	3.01	3.03	3.12	3.07	0.06
**500**	2.79	2.26	2.62	2.56	0.27
**200**	1.96	1.96	2.67	2.20	0.41
**Genotype 4**					
**1,000,000**	5.51	5.50	5.51	5.51	0
**100,000**	4.62	4.60	4.60	4.61	0.02
**10,000**	3.67	3.59	4.44	3.90	0.47
**1000**	2.79	2.85	2.74	2.79	0.06
**500**	2.31	2.33	2.09	2.24	0.14
**200**	0	0	2.05	NA	NA
